# Intra-Urban Variation of Intimate Partner Violence Against Women and Men in Kenya: Evidence from the 2014 Kenya Demographic and Health Survey

**DOI:** 10.1177/08862605221120893

**Published:** 2022-09-05

**Authors:** Beate Ringwald, Rachel Tolhurst, Miriam Taegtmeyer, Lina Digolo, Grace Gichuna, Mwangi Michael Gaitho, Penelope A. Phillips–Howard, Lilian Otiso, Emanuele Giorgi

**Affiliations:** 1 Liverpool School of Tropical Medicine, UK; 2 The Prevention Collaborative, Nairobi, Kenya; 3 LVCT Health, Nairobi, Kenya; 4 Lancaster University, UK

**Keywords:** intimate partner violence, domestic violence, urban health, slum, informal settlement, Kenya

## Abstract

Although urban areas are diverse and urban inequities are well documented, surveys commonly differentiate intimate partner violence (IPV) rates only by urban versus rural residence. This study compared rates of current IPV victimization among women and men by urban residence (informal and formal settlements). Data from the 2014 Kenya Demographic and Health Survey, consisting of an ever-married sample of 1,613 women (age 15–49 years) and 1,321 men (age 15–54 years), were analyzed. Multilevel logistic regression was applied to female and male data separately to quantify the associations between residence and any current IPV while controlling for regional variation and other factors. Results show gendered patterns of intra-urban variation in IPV occurrence, with the greatest burden of IPV identified among women in informal settlements (across all types of violence). Unadjusted analyses suggest residing in informal settlements is associated with any current IPV against women, but not men, compared with their counterparts in formal urban settlements. This correlation is not statistically significant when adjusting for women’s education level in multivariate analysis. In addition, reporting father beat mother, use of current physical violence against partner, partner’s alcohol use, and marital status are associated with any current IPV against women and men. IPV gets marginal attention in urban violence and urban health research, and our results highlight the importance of spatially disaggregate IPV data—beyond the rural-urban divide—to inform policy and programming. Future research may utilize intersectional and syndemic approaches to investigate the complexity of IPV and clustering with other forms of violence and other health issues in different urban settings, especially among marginalized residents in informal urban settings.

## Introduction

Intimate partner violence (IPV) is a widespread health, wellbeing, equity, and justice problem (Muluneh et al., 2020; World Health Organization [WHO] et al., 2013). IPV is common in Kenya where one in two women and one in four men report emotional, physical, and/or sexual IPV experience (also referred to as victimization) during their lifetime, including one in three women and one in five men experiencing “current IPV” defined as IPV experienced in the last 12 months (Ellsberg & Heise, 2005; Kenya National Bureau of Statistics [KNBS] et al., 2015a). Current IPV prevalence varies across the country: among women, from 37% in the Western region and 35% in Nairobi to 6% in the North-Eastern region, and from 11% in Nairobi to 3% in the North-Eastern region among men (KNBS et al., 2015a).

Multi-country studies on violence against women suggest IPV risk is greater in rural than urban areas (Coll et al., 2020; García-Moreno et al., 2005). In contrast, prevalence estimates of current physical and/or sexual IPV experience are comparable between urban and rural populations in Kenya (women: 25% vs. 26%; men: 8% vs. 7%) (KNBS et al., 2015a). While urban areas are diverse spaces, national surveys investigating IPV, such as the Kenya Demographic and Health Survey (KDHS), do not disaggregate beyond the conventional urban-rural divide. Limiting IPV prevalence estimates to “urban” and “rural” residence hides variation and inequalities within settings, and opportunities for targeted interventions may be missed.

### Defining Informal Urban Settlements

Disaggregating urban data at intra-city level is common in global reports on urban health and living (UN-Habitat, 2006; WHO, 2016). Education, employment, housing, and safety inequalities are well documented in cities, including Kenya’s capital city Nairobi (African Population and Health Research Center [APHRC], 2014). Fifteen million of the estimated 47.6 million Kenyans reside in urban areas (KNBS, 2019b), and more than half (56%) live in slums (UN-Habitat, 2016). UN-Habitat defines an urban slum household as one lacking access to improved water and sanitation; security of tenure; durability of housing; and sufficient living area (UN-Habitat, 2016). Other definitions cover the lack of basic services such as education, electricity, and transportation (Cities Alliance, n.d.; Habitat for Humanity Great Britain, n.d.). We use the term “informal settlement” to acknowledge the absence of essential services as an identifying characteristic since the term “slum” has derogatory connotations (Lines & Makau, 2017).

### Compounded Inequalities in Informal Urban Settlements

People living in Kenya’s informal settlements face challenges ranging from insecurity and unemployment to unmet needs for family planning and contraception (APHRC, 2014). Poor health outcomes, including high rates of HIV (Madise et al., 2012) and teenage pregnancy (APHRC, 2014), are connected to poverty, marginalization, and limited access to quality health services in these areas (Zulu et al., 2011). IPV gets marginal attention in global urban reports which tend to focus on insecurity, crime, and violence more broadly. However, IPV studies conducted among women (Ringwald et al., 2020; Orindi et al., 2020; Swart, 2012) and men (Ringwald et al., 2020) in informal settlements in Nairobi reported rates of IPV above KDHS urban prevalence estimates. Contrary to widely reported gender gaps, IPV studies in informal settlements in Nairobi and Dar-es-salaam found comparable rates among women and men (Ringwald et al., 2020; Mulawa et al., 2018). The impact of IPV in informal settlements may be particularly grave due to the economic burden of IPV-related harm on survivors, families, and communities (National Gender and Equality Commission, 2016).

### Conceptualizing IPV

The ecological framework is used globally and in sub-Saharan Africa to conceptualize male-to-female IPV and takes into account gender inequality as underlying driver (Raising Voices & African Women’s Development Fund, 2019). According to the model, a complex interplay of factors across levels of the social ecology—from individual, partner, relationship, community to societal levels—causes IPV (Heise, 2011). Frameworks conceptualizing female-to-male IPV locate it within bilateral couple violence (Johnson & Leone, 2005) and as a response to male-to-female IPV (Swan et al., 2008), although there is limited literature on this from sub-Saharan Africa. The ecological model has the potential to accommodate a variety of factors and their interplay in relation to women and men’s IPV experiences.

### IPV Risk Factors Reported in Kenya

IPV research in Kenya has focused mainly on women to date and identifies various individual and partner-level risk factors. Women’s education reduces and poverty increases their risk of experiencing IPV (Abuya et al., 2012; Bamiwuye & Odimegwu, 2014), while men’s unemployment enhances their IPV risk (Gateri et al., 2021). Women who are married or cohabiting (Burmen et al., 2018; Gust et al., 2017) and formerly married men; women and men who witnessed IPV between parents (i.e., father beat mother) during childhood (Ringwald et al., 2020); women and men who also report to be perpetrators of IPV (Ringwald et al., 2020); and women whose partners use alcohol or drugs (Gust et al., 2017; Owaka et al., 2017) are disproportionally affected.

Evidence on community-level IPV risk factors is sparse. Neighborhood effects on IPV have mainly been studied in high-income countries and rarely in sub-Saharan Africa (Alderton et al., 2020). A study on female-to-male IPV suggests economic and social environments in rural Kenya trigger marital conflicts and IPV against men (Gateri et al., 2021). Community norms and deprivation are known to amplify male-to-female IPV risk. One in three people justify wife beating in urban Kenya (KNBS et al., 2015a) and inequitable gender norms and patriarchal culture condone men’s use of violence against women as a means of discipline, maintaining male dominance and control (Gillum et al., 2018; Hatcher et al., 2013). Furthermore, multiple intersecting disadvantages based on gender, class, socio-economic status, and education shape women’s experience of IPV since patriarchy is intertwined with other systems of oppression (Coalition of Feminists for Social Change, 2018). For example, intersecting gender and economic inequalities influence experiences of poor women in Kenya and those who depend on their partners economically (Gillum et al., 2018; Hatcher et al., 2013).

### Kenya’s Commitments Toward Eliminating IPV

Kenya is committed to “*significantly reducing all forms of violence*” (Sustainable Development Goal 16) and “*eliminating all forms of violence against all women and girls*” (SDG 5). These commitments are evidenced in its Constitution (2010), the Protection Against Domestic Violence Act (2015), and the Sexual Offences Act (2006). The Ministry responsible for gender coordinates the multi-sectoral response laid out in national policy (Ministry of Devolution and Planning, 2014). Several toll-free hotlines provide IPV survivors with information and referral. In 2021, Kenya renewed its commitments to scaling up one-stop victim support “Policare” centers in all counties (Kenya Police Service, n.d.); and integrating medical, legal, and psychological gender-based violence services into the universal health coverage program (Government of Kenya, 2021).

### Research Problem, Aim, and Hypotheses

Although the consequences of IPV in informal urban settlements may be particularly grave, the comparative burden of IPV in informal urban settlements in Kenya has not been reliably quantified. Evidence on the burden of IPV in informal urban settlements has mainly been generated through research in Nairobi’s informal settlements; studies lacked comparators in other urban areas; and research often involved small numbers of respondents. Consequently, the results are not necessarily generalizable to informal settlements countrywide and do not quantify potential differences in IPV prevalence in informal and formal urban settlements.

**Figure 1.**
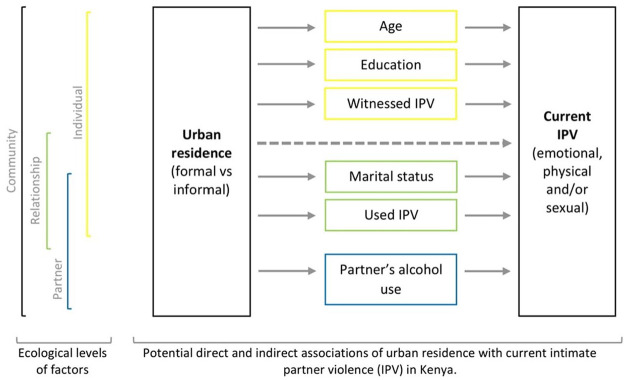
Conceptual framework of associations between urban residence and intimate partner violence (IPV).

This study aimed to compare rates of current IPV experience among women and men by urban residence (informal and formal settlements); for this, we used data from the 2014 KDHS. The analysis is based on an ecological model to investigate direct associations between urban residence and IPV and indirect associations mitigated by individual, relationship, and partner factors, as shown in Figure 1. Based on the review of the literature, we tested the following hypotheses:

Hypothesis 1: Female and male prevalence of any current IPV is higher in informal than in formal urban settlements.
Hypothesis 2: Female and male prevalence of any current IPV in informal urban settlements is comparable.
Hypothesis 3: Informal urban residence is directly correlated with any current IPV against women and men, even after adjusting for individual, relationship, and partner factors.

## Methods

This study is nested within the ARISE Hub (URL: http://www.ariseconsortium.org/). ARISE is a research consortium of partners in Bangladesh, India, Kenya, Sierra Leone and the UK working towards catalysing change in approaches to enhancing accountability and improving the health and wellbeing of poor, marginalised people living in informal urban settlements. Within this research partnership, European and Kenyan researchers at the Liverpool School of Tropical Medicine (LSTM) and LVCT Health jointly conducted this analysis.

### Dataset and Sample

This study used data from the 2014 KHDS, a cross-sectional household survey designed to produce nationally representative estimates for urban and rural areas of a wide range of health and socio-economic indicators (KNBS et al., 2015a). Through a two-stage sampling design, 39,679 households were selected for the survey. First, enumeration areas (*n* = 1,612, including 617 urban clusters) were randomly selected from a national sampling frame comprising 92 sampling strata (45 urban and 47 rural strata). Then, an equal number of households was sampled within each cluster. Individual interviews were conducted in person among women (age 15–49 years) and men (age 15–54 years) from May to November 2014. Questionnaires were administered in English and 16 other Kenyan languages. Details on survey design and methods can be found in the 2014 KDHS report (KNBS et al., 2015a). We obtained 2014 KDHS datasets [dataset] (KNBS et al., 2015b) through application to the DHS Program.

Of the 31,079 female and 12,819 male respondents, 5,657 females and 4,962 males were interviewed on domestic violence. Married, cohabiting, separated, divorced, and widowed respondents (4,519 females and 3,268 males) were asked about IPV. Since our analysis focused on the variation of IPV within urban areas, we excluded records from rural areas and selected observations among 1,644 female and 1,331 male respondents residing in urban areas.

Observations with missing values of relevant variables were excluded from the analysis (31 females, 10 males). These included missing values on IPV experience (two females, two males); missing or inconsistent data on the primary source of drinking water and toilet facility (24 females) and electricity (one male), which were used for approximating the type of residence; and missing values among other explanatory variables (five females, seven males). We retained 1,613 female and 1,312 male observations with complete data in the analytic sample.

### Outcome Variable

Our primary outcome was current IPV experience as measured by the KDHS domestic violence module, an adaption of the revised Conflict Tactics Scale (Kishor & Johnson, 2004) first validated for use with females and males (Straus et al., 1996). The questions assess concrete acts of violence by a spouse/partner involving emotional violence, including (a) humiliated in front of others; (b) threatened to hurt or harm respondent or someone close; or (c) insulted or made feel bad; physical violence, including (a) pushed, shook or threw something; (b) slapped; (c) twisted arm or pulled hair; (d) punched with fist or something that could hurt; (e) kicked, dragged, or beat up; (f) tried to choke or burn; or (g) threatened or attacked with knife, gun, or another weapon; and sexual violence including (a) forced to have sexual intercourse when not wanted; (b) physically forced to perform any other sexual acts when not wanted; or (c) forced respondent with threats or in any other way to perform sexual acts spouse when not wanted.

A binary variable was coded for each act of current violence (0 = never or not in the last 12 months, 1 = often or sometimes in the past 12 months). IPV was classified as emotional, physical, and sexual, and a combination of these summarized as “any IPV.” Composite variables were coded “1” when at least one listed act of violence occurred in the past 12 months.

### Spatial Variables

#### Residence

In line with the UN-Habitat definition, we used household-level housing indicators as proxies for defining the type of urban residence, our primary explanatory variable. As introduced by Zulu et al. (2002) and applied by Madise et al. (2012), the urban residence variable considered respondents’ household’s access to electricity, improved sanitation, and improved water (0 = no, 1 = yes). Improved sanitation was identified if a household had a flush toilet (including flushed to a piped sewer system, septic tank, pit latrine, or unspecified); and improved water was identified if a household had water piped into the dwelling, yard, or plot. Residence in an informal settlement was defined as the simultaneous absence of electricity, improved sanitation, and improved water (informal = 0). Residence in a formal settlement was defined as the simultaneous presence of the three facilities (formal = 3). Households reporting one or two facilities were assigned as “intermediate” (intermediate = 1 or 2).

#### Province

Urban communities vary across the country, including in size and population density (KNBS, 2019a). We opted to include province (*n* = 8), the former administrative unit in Kenya, instead of counties (*n* = 47), as some counties had too few observations.

### Other Variables

#### Individual characteristics

We describe the female and male samples by age (coded as single years of age), wealth (reported as wealth quintiles derived through household asset index approach; 1 = poorest, 2 = poorer, 3 = middle, 4 = richer, 5 = richest), and education level (0 = no schooling, 1 = primary, 2 = secondary, 3 = higher education).

#### Marital status

Respondents’ marital status was treated as a categorical variable (1 = married, 2 = cohabiting, 3 = separated, 4 = divorced, 5 = widowed).

#### Witnessed father beat mother

Within the domestic violence module, respondents reported if their father ever beat their mother (0 = no, 1 = yes, 2 = don’t know). Respondents were not asked if the mother ever beat the father.

#### Current use of physical violence against partner

Based on a single question, “Have you ever hit, slapped, kicked, or done anything else to physically hurt your (last) (spouse/partner) at times when he/she was not already beating or physically hurting you?” a binary variable was coded (0 = never or not in the last 12 months, 1 = sometimes or often in the past 12 months).

#### Partner’s alcohol use

Respondents were asked if their partner drank alcohol and if those who did so got drunk never, sometimes, or often (0 = does not drink or never gets drunk, 1 = gets drunk sometimes, 2 = gets drunk often). Since few male respondents reported their partner drank alcohol, a binary response was retained (0 = does not drink alcohol, 1 = drinks alcohol).

### Statistical Analysis

Our analysis was stratified by sex as standard in other studies (Ringwald et al., 2020; Papas et al., 2017) to account for differences in IPV experience between women and men. The description of the female (*n* = 1,613) and male (*n* = 1,321) samples was stratified by residence. We estimated prevalence of different types of IPV experience, stratified by sex and residence.

We used multilevel logistic regression to quantify the associations between residence and any current IPV. The analysis was conducted with current IPV experience as the outcome and those who reported having never experienced IPV as the reference group (Vyas & Heise, 2016). Observations with lifetime but not current IPV experience among females (*n* = 184) and males (*n* = 76) were excluded, yielding an analytic sample of 1,429 female and 1,245 male observations for analysis. We formulated binomial mixed-effects models with fixed effects at the individual level and random effects at the cluster level (i.e., provinces) because we observed significant variation in IPV prevalence across provinces (KNBS et al., 2015a). Random effects at the cluster level account for overdispersion and variation unexplained by other covariates. The fixed effects of individual-level parameters are constant over all provinces.

We modeled the commonly used composite measure “any IPV” experience (Memiah et al., 2018) and assessed its correlations with residence. We conducted bivariate analysis (Model 1) and multivariate analysis (Model 2), adjusting for factors linked to IPV in previous studies. Models adjust for respondent’s education level, marital status, having witnessed father beat mother, and use of physical violence against partner, partner’s alcohol use. We sought to minimize model complexity so as not to destabilize models. Therefore, age was excluded after preliminary analysis showed no correlation between age (including on log-scale) and current IPV experience. In addition, categorical variables were recoded, collapsing levels when having few observations or observing similarities between levels in preliminary analysis (i.e., female education level: 0 = no schooling, 1 = primary or secondary, 2 = higher education; male education level: 1 = no schooling, primary or secondary, 2 = higher education; female marital status: 1 = married, 2 = cohabiting, 3 = separated or divorced, 4 = widowed; male marital status: 1 = married, 2 = cohabiting, 3 = separated, divorced, or widowed). The KDHS household wealth index is based on household-level housing indicators. Since we used some of these indicators to approximate residence, the analysis did not adjust for household wealth. We followed the standard practice for analysis of subsamples of DHS data and did not apply sample weights (Durevall & Lindskog, 2015). We report fixed effects with odds ratios (*OR*), 95% confidence intervals (CI) and respective *p*-values. Statistical analysis was done in RStudio (Version 1.3.1093).

### Ethical Considerations

The institutional review board of ICF International reviewed and approved the DHS-7 used in the current study (ICF Project Number: 132989.0.000). We sought and received formal permission from DHS Program, ICF, to use the 2014 KDHS datasets. Our study (registration number 19-067) did not require formal review by LSTM Research Ethics Committee as datasets are publicly available and fully anonymized. The authors provided R-codes to replicate the study upon request.

The KDHS 2014 was conducted following the WHO’s ethical and safety recommendations for research on domestic violence (WHO, 2001): Only one person per household was interviewed on domestic violence to ensure confidentiality; additional consent was obtained for the domestic violence questionnaire; violence questions were administered when no other person was present (except for small children); and respondents were given contact details for domestic violence service centers (KNBS et al., 2015a).

## Results

One-third of women (38%) and men (33%) were identified as residents of informal settlements; about one in eight women (13%) and men (15%) were identified as residing in formal settlements; half of the women (49%) and men (51%) were assigned to the “intermediate” residence class (Supplemental Appendix A). The median age is 29 years in the female sample and 35 years in the male sample. While most women (89.7%) and men (96.1%) have some level of schooling, educational attainment is lower in informal settlements than in other settlements. For example, in informal settlements, women are 15 times and men six times less likely to have accomplished higher education than counterparts in formal settlements. Overall, about two-thirds of urban residents are in the top two wealth quintiles. Urban poor reside almost exclusively in informal settlements, and only a tiny minority live in formal and intermediate settlements. Most women (77.6%) and men (88.4%) are married. More men (44.3%) than women (35.6%) recall their father beating their mother. More men (95.2%) than women (67.7%) have a partner who does not drink alcohol or get drunk. One in 10 women state their partner gets drunk “often,” while two in 10 state he gets drunk “sometimes.” Very few women (2.6%) and some men (13.0%) used physical violence against a partner in the past 12 months.

### Prevalence of Any Current IPV Experience and Overlap With Current Use of IPV

**Figure 2.**
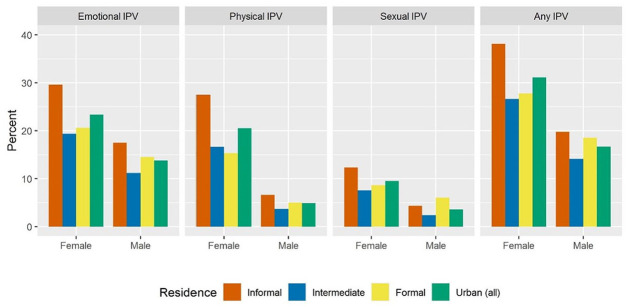
Prevalence of current intimate partner violence (IPV) among female (age 15–49 years) and male (age 15–54 years) residents in urban areas of Kenya by type of IPV (columns) and residence (2014).

Figure 2 shows prevalence estimates of any current IPV against women and men in urban areas. Across all types of current IPV experienced, prevalence is higher among women than men, including in informal urban settlements where 38% women and 20% men reported any current IPV, mainly emotional IPV (30% vs. 18%), followed by physical IPV (27% vs. 7%) and sexual IPV (12% vs. 4%). Rates of IPV experience among women in informal settlements are above urban averages (e.g., any current IPV: 38% vs. 31%) and higher than those in formal settlements (e.g., any current IPV: 38% vs. 28%). In contrast, rates of IPV experience among men are comparable between informal and formal settlements (any current IPV: 20% vs. 19%).

**Figure 3.**
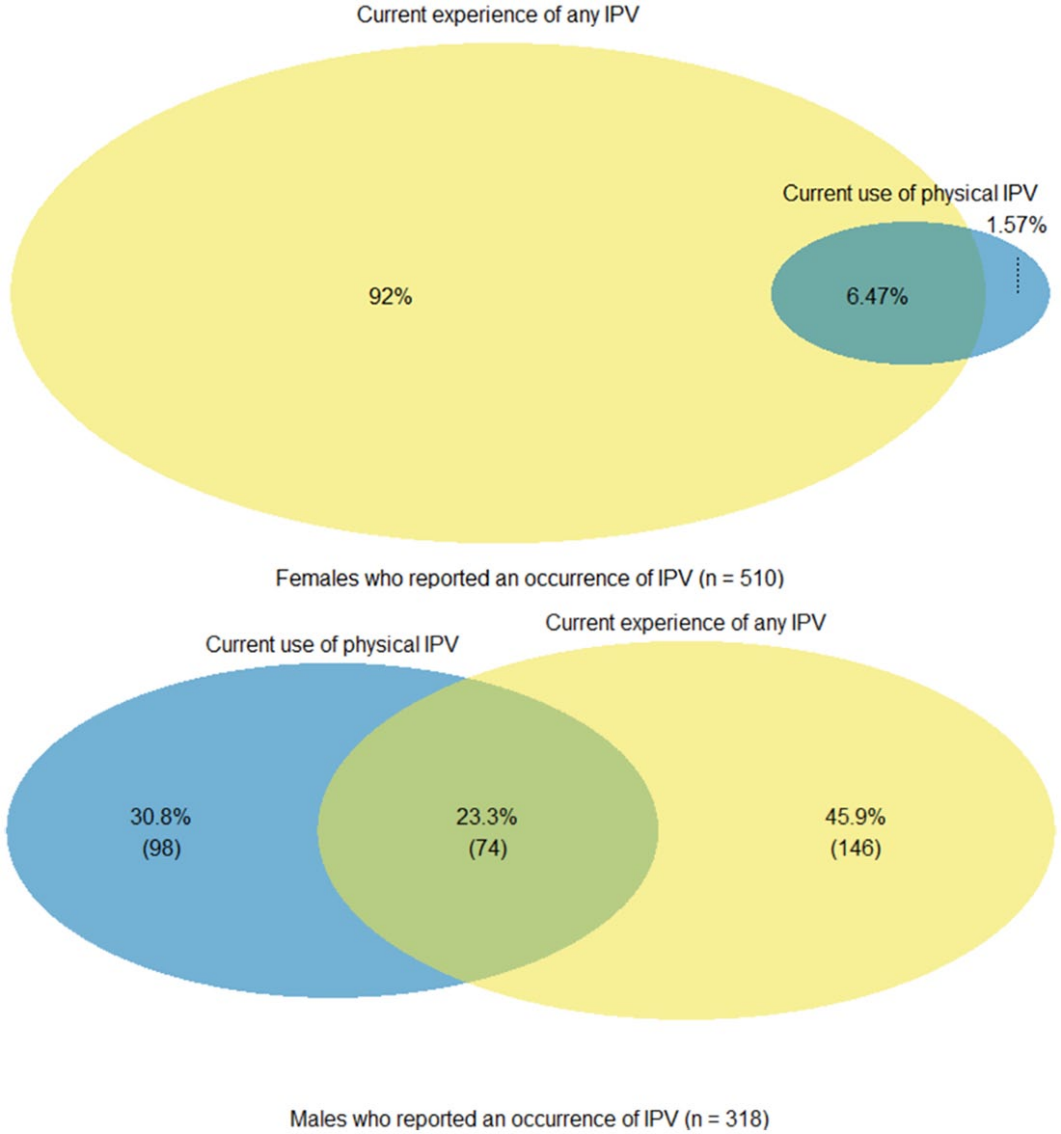
Overlap between current experience of any intimate partner violence (IPV) and current use of physical violence against partner among female residents (age 15–49 years) (upper panel) and male residents (age 15–54 years) (lower panel) in urban areas of Kenya who reported an occurrence of IPV in the past 12 months (2014).

Figure 3 shows the overlap between current experience of any IPV and current use of physical violence against partner. According to the data of current IPV, most women (68%) and men (76%) do not experience any IPV nor use physical violence against a partner. Patterns of the overlap vary: Nearly all women (92.0%) and about half of men (45.9%) state experiencing any current IPV without using physical violence against partner (Figure 3). Current use of physical violence against a partner without current victimization is rare among women (1.6%) but more common among men (30.8%). Concurrent experience of any current IPV and use of current physical violence against a partner is less common among women (6.5%) than men (23.3%).

### Correlations Between Type of Residence and IPV

**Figure 4.**
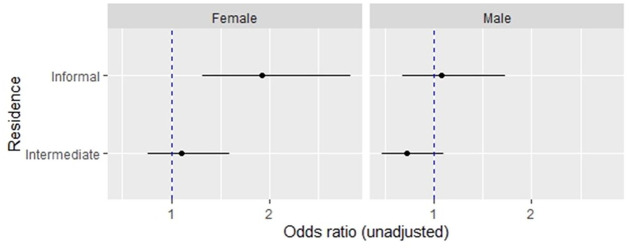
Unadjusted estimates from binomial mixed-effects models for any current intimate partner violence against women (age 15–49 years) and men (age 15–54 years) in urban areas in Kenya (2014).

Figure 4 shows the unadjusted estimates from binomial mixed-effects models for any current IPV experience. Estimates suggest a statistically significant association between residing in informal urban settlements and any current IPV experience among women (*OR* 1.92 [95% CI: 1.31, 2.83]), unlike among men (*OR* 1.08 [95% CI: 0.68, 1.73]), when compared with those residing in formal urban settlements. For tabulation of model estimates, see Supplemental Appendix B.

**Figure 5.**
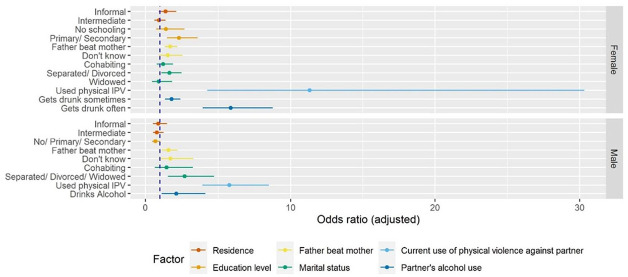
Adjusted estimates from binomial mixed-effects models for any current intimate partner violence against women (age 15–49 years) and men (age 15–54 years) in urban areas in Kenya (2014).

Figure 5 shows the adjusted estimates from binomial mixed-effects models for any current IPV experience. Female data suggest correlation between residing in informal settlements and any current IPV experience among women is not statistically significant (adjusted OR [aOR] 1.39 [95% CI: 0.91, 2.13]) when adjusting for individual, relationship, and partner factors. The odds of any current IPV experience are higher among women with primary or secondary education (aOR 2.31 [95% CI: 1.49, 3.57]) when compared to those with higher education. Additional analyses (shown in Supplemental Appendix C) indicate women’s education level mediates the correlation between informal settlement residence and any current IPV experience. Other factors at the individual, relationship, and partner levels do not change estimates of this correlation.

The odds of any current IPV experience are higher among women and men who witnessed father beat mother (aOR 1.71 [95% CI: 1.33, 2.20]) and (aOR 1.59 [95% CI: 1.14, 2.22]) when compared with those who did not. Female data suggest several relationship and partner factors correlate with any current IPV experience. These include being separated or divorced (aOR 1.65 [95% CI: 1.1, 2.49]), women’s current use of physical violence against their partner (aOR 11.32 [95% CI: 4.23, 30.34]), and having a partner who gets drunk sometimes (aOR1.81 [95% CI: 1.35, 2.42]) and often (aOR 5.86 [95% CI: 3.92, 8.77]). Male data similarly suggest relationship and partner factors are correlated with any current IPV experience. These include being separated, divorced, or widowed (aOR 2.71 [95% CI: 1.56, 4.71]), men’s current use of physical violence against their partner (aOR 5.76 [95% CI: 3.91, 8.49]), and having a partner who uses alcohol (aOR 2.14 [95% CI: 1.11, 4.11]). Tabulation of unadjusted and adjusted model estimates are shown in Supplemental Appendices B to D. Estimates of the variation of the random effect of the cluster variable “Province” are shown in Supplemental Appendix E.

## Discussion

We set out to compare IPV against women and men by urban residence. Our analysis shows women reported higher rates of IPV experience than men across all urban settlements. Women in informal urban settlements experienced the highest rates of IPV across all types of violence, supporting Hypothesis 1 for female data (i.e., higher prevalence of any current IPV in informal than formal urban settlements). These results confirm high rates of IPV experience observed among women in informal settlements in Nairobi (Ringwald et al., 2020; Orindi et al., 2020; Swart, 2012). The estimated prevalence of any current IPV experience among men is comparable to the pooled estimate of female-to-male IPV prevalence in Africa (Lindstrøm, 2018). However, contrary to our Hypotheses 1 and 3, male results indicate rates of IPV against men are high in informal and formal urban settlements and men’s residence is not associated with experience of any current IPV. Furthermore, data show evidence against Hypothesis 2 as women in informal settlements experience a greater burden of current IPV than men across all types of violence. Observed gender differences in IPV prevalence are consistent with results of a national survey in South Africa (Gass et al., 2011) but contrast with results from research in informal settlements in Nairobi and Dar-es-salaam reporting comparable rates of IPV experience among women and men (Ringwald et al., 2020; Mulawa et al., 2018). Our results are based on female and male samples representative of urban areas in Kenya and show high burdens of IPV apply to informal settlements countrywide.

The results highlight the compounded disadvantage of women in informal urban settlements as they bear the brunt of IPV. In unadjusted analysis, informal settlement residence, identified by the absence of household amenities, is significantly associated with IPV against women. Studies conducted in African informal urban settlements reported women’s increased risk of domestic and non-partner violence due to housing deprivation and lack of infrastructure (Barchi & Winter, 2019; Meth, 2017; Pommells et al., 2018; Shiras et al., 2018). Contrary to our expectation, however, female data do not support Hypothesis 3 (i.e., direct association between informal urban residence and any current IPV after adjusting for control variables). When models adjust for women’s individual, relationship, and partner factors, informal urban residence is not significantly associated with any current IPV experience.

Our analysis suggests women’s education level plays a role. It is plausible the benefits of education (better job opportunities, increased disposable income, enhanced access to knowledge, more equitable gender attitudes, and greater control over intimate partnerships) mediate the association and reduce women’s IPV risk (Bates et al., 2004; Brooks et al., 2019; Gillum et al., 2018; Mugoya et al., 2015). Free public primary schools are, however, scarce in informal settlements, and families rely on small private “non-formal” schools for primary education (Oketch et al., 2010) facing a “poverty penalty”; they pay more to receive inferior educational services (Malenya, 2020). Since employment and income opportunities are limited, financial constraints force children (girls more often than boys) to drop out of school (APHRC, 2014). Children from informal settlements have disproportionally low chances of joining public secondary schools (Ohba, 2013), and girls face additional barriers. These include sexual harassment; challenges paying for sanitary products and managing menstruation in schools (Girod et al., 2017); and unintended pregnancies (Beguy et al., 2014) which hinder school re-entry (Walgwe et al., 2016). Women in Kenya are underrepresented in tertiary learning institutions (Ministry of Devolution and Planning, 2016), with additional barriers to accessing higher education for those residing in informal settlements.

To our surprise, men in formal and informal settlements experience comparable rates of IPV. Since a study in rural Kenya had suggested economic, physical, and social environments shape individual risk factors of female-to-male IPV (Gateri et al., 2021), we expected to find evidence for correlations of IPV with residence as proxy for community factors. Results from rural Kenya suggest women’s frustration and desire to control and men’s alcohol use and infidelity trigger female-to-male IPV (Gateri et al., 2021). However, we could not investigate these factors, nor ascertain whether correlations between current IPV experience and current use of IPV, observed among women and men, constituted bilateral partner violence, acts of self-defense, or response to partner’s use of IPV. Observed correlations between experience and use of current IPV are consistent with studies that investigated IPV victimization and perpetration in Kenya and Tanzania (Ringwald et al., 2020; Mulawa et al., 2018). Media coverage of female-to-male IPV frequently gets sensational attention in Kenya (King’ori & Bitrus-Ojiambo, 2017). Although the question “what about men?” is often raised (e.g., Osindo et al., 2018), there is a paucity of high-quality studies on female-to-male IPV in Kenya to date.

Female and male data indicate partner’s alcohol use strongly correlates with any current IPV—independent of the respondent’s residence and other control variables. On the one hand, these results resonate with a multi-country study that found links between alcohol and IPV for both women and men (Graham et al., 2018). On the other hand, alcohol does not fully explain the link, since partners are not always intoxicated when abusive or abusive when intoxicated (Kelly, 2011). Expanding our focus to the interplay of community and individual factors, research in urban Tanzania suggests that the heavy presence of alcohol-selling outlets signals social acceptance of drinking (Ibitoye et al., 2019). High density of alcohol outlets can create environments where alcohol use and IPV risk mutually reinforce each other; with easy access to alcohol stimulating patterns of drinking, while triggering IPV; and alcohol outlets providing opportunities for forming groups and practices that reinforce IPV-related attitudes (Cunradi, 2010). The widespread production and consumption of traditional homebrew in Kenya complicates efforts to prevent harm related to excessive alcohol use (Mkuu et al., 2019).

Our results suggest IPV deserves greater attention in the fields of urban health and urban violence. Compared to crime and other forms of violence, IPV plays a marginal role in these discussions. Studies from sub-Saharan Africa found IPV does not occur in isolation but overlaps with other forms of violence: violence against children (Namy et al., 2017); domestic violence and collective political violence (The World Bank, 2011). Experience and perpetration of different forms of violence in urban areas are interlinked, with blurred lines between different expressions of violence (The World Bank, 2011). Consequently, those experiencing a disproportionally great burden of IPV, including people in informal settlements, are likely to be exposed to other forms of violence and, subsequently, at risk of poor health outcomes. While urbanization provides opportunities that are potentially protective against IPV, the pressures of urban living contribute to a context where IPV can flourish (McIlwaine, 2013).

Despite Kenya’s progress in establishing legal and policy frameworks, limited coordination among sectors and service providers; limited financial and human resources and equipment; lack of knowledge among service providers; and flawed evidence collection impede enforcement (Ajema et al., 2011; Kilonzo et al., 2009; Wangamati et al., 2021) and successful prosecution (Committee on the Elimination of Discrimination against Women [CEDAW], 2017). Cases of IPV are underreported due to financial barriers and fear of an unsupportive or discriminatory response from service providers (Fernandes et al., 2020). For example, service providers may illegally charge women in informal settlements for reporting forms (CEDAW, 2017). Moreover, access to services is by limited awareness of own rights, lack of knowledge of existing services, acceptance of IPV, and stigma (Fernandes et al., 2020). IPV survivors’ rates of help-seeking remain low (KNBS et al., 2015a), especially among men (Gatuguta et al., 2018).

### Recommendations

We recommend more attention is given to addressing interconnecting challenges and identifying integrated approaches to preventing IPV and addressing related challenges. IPV research needs an more intersectoral lens. Future studies should assess how types of urban residence, gender, and other axes of disadvantage (such as wealth, (dis)ability, or ethnicity) intersect in shaping IPV risk in greater depth. A syndemic approach (Singer, 1996) may provide a suitable lens for exploring the complex nature of IPV; illuminating processes through which IPV is interconnected both with other social and health conditions and the environment.

We further recommend actions to advance the collection of better-disaggregated urban IPV data. The Nairobi Urban Health and Demographic Surveillance System collects data on various health indicators in two informal settlements in Nairobi and provides a platform for providing reliable IPV prevalence estimates if a domestic violence module was added to the survey. Given a high concentration of research in informal settlements in Kenya’s capital city, future research should be conducted in urban centers other than Nairobi. Extensive cross-sectional surveys like the DHS would benefit from disaggregating urban residence with categories relevant to the context. At a minimum, a distinction between informal and formal settlements should be made in Kenya.

The KDHS classified respondents as female and male and assumed heterosexual relationships. Hence, the survey was not equipped to involve and report on intersex and non-binary people and same-sex relationships. Gender and sexual minorities in Kenya may face high risk to IPV, as observed in Tanzania (Mgopa et al., 2017), given the legal and social marginalization. Alternative study designs are needed to document their burdens and experiences of IPV.

### Limitations

This study used household-level housing indicators as a proxy to determine type of residence. A third of urban respondents were identified as residing in informal settlements, yet countrywide more than half of urban residents live in informal settlements (UN-Habitat, 2016). We cannot rule out some of those assigned to the “intermediate” class actually living in informal settlements in households with access to amenities (APHRC, 2014). It is possible some people living in informal settlements were excluded from the 2014 KDHS because its sampling frame was aligned with administrative boundaries outside of rapidly emerging informal settlements (Chumo et al., 2021). Given that IPV prevalence was high in informal settlements and people in informal settlements were potentially underrepresented, we hypothesize average urban IPV prevalence was underestimated. In addition, our results suggest disparate urban distributions of household wealth. Since women’s socio-economic status is protective against IPV (Vyas & Watts, 2009), adjustments for socio-economic status would have been useful, but was not possible.

IPV estimates are based on presence in the household and on respondents’ self-reported information, both of which may have introduced bias. Firstly, the response rate was greater among women (95%) than men (87%) in urban households selected for the full questionnaires, which included the domestic violence module, mainly due to absence from home (KNBS et al., 2015a). Secondly, we cannot rule out underreporting due to recall and social desirability biases in contexts where IPV is normalized and stigmatized. The 2014 KDHS applied measures to maximize participation, minimize bias, and enhance data quality: Questionnaires were administered in various languages; same sex interviewers received training on asking sensitive questions and building rapport; IPV was measured with a validated research instrument; and respondents were asked about a wide range of violent acts providing respondents with multiple reliable opportunities to recall and disclose IPV experience.

Finally, we used 2014 KDHS data not collected for the purpose of our study. The cross-sectional nature of the data limits our ability to draw causal inferences for the identified associations while situating results within the literature. Although recommended (Merson et al., 2018), we were not able to directly involve 2014 KDHS data collectors and researchers but did conduct our study within an established partnership of European and Kenyan researchers.

## Conclusion

This study quantified intra-urban variation of IPV experience in Kenya, highlighting the need to spatially disaggregate IPV data beyond the rural-urban divide. Multilevel logistic regression analysis aided in identifying associations of individual, relationship, and partner factors with any current IPV experience, while the ecological model assisted in interpreting and contextualizing results. High rates of IPV experience in informal settlements, especially among women, suggest work on urban violence and urban health ought to pay greater attention to IPV. Future research is recommended to evaluate the impact of gendered urbanization processes on IPV in greater depth; there is potential to utilize intersectional and syndemic approaches to advance understanding about the complexities and interconnectedness of IPV and identify integrated approaches to address IPV and related challenges in diverse urban settings.

## Supplemental Material

sj-pdf-1-jiv-10.1177_08862605221120893 – Supplemental material for Intra-Urban Variation of Intimate Partner Violence Against Women and Men in Kenya: Evidence from the 2014 Kenya Demographic and Health Survey Supplemental material, sj-pdf-1-jiv-10.1177_08862605221120893 for Intra-Urban Variation of Intimate Partner Violence Against Women and Men in Kenya: Evidence from the 2014 Kenya Demographic and Health Survey by Beate Ringwald, Rachel Tolhurst, Miriam Taegtmeyer, Lina Digolo, Grace Gichuna, Mwangi Michael Gaitho, Penelope A. Phillips–Howard, Lilian Otiso and Emanuele Giorgi in Journal of Interpersonal Violence

sj-pdf-2-jiv-10.1177_08862605221120893 – Supplemental material for Intra-Urban Variation of Intimate Partner Violence Against Women and Men in Kenya: Evidence from the 2014 Kenya Demographic and Health Survey Supplemental material, sj-pdf-2-jiv-10.1177_08862605221120893 for Intra-Urban Variation of Intimate Partner Violence Against Women and Men in Kenya: Evidence from the 2014 Kenya Demographic and Health Survey by Beate Ringwald, Rachel Tolhurst, Miriam Taegtmeyer, Lina Digolo, Grace Gichuna, Mwangi Michael Gaitho, Penelope A. Phillips–Howard, Lilian Otiso and Emanuele Giorgi in Journal of Interpersonal Violence

sj-pdf-3-jiv-10.1177_08862605221120893 – Supplemental material for Intra-Urban Variation of Intimate Partner Violence Against Women and Men in Kenya: Evidence from the 2014 Kenya Demographic and Health Survey Supplemental material, sj-pdf-3-jiv-10.1177_08862605221120893 for Intra-Urban Variation of Intimate Partner Violence Against Women and Men in Kenya: Evidence from the 2014 Kenya Demographic and Health Survey by Beate Ringwald, Rachel Tolhurst, Miriam Taegtmeyer, Lina Digolo, Grace Gichuna, Mwangi Michael Gaitho, Penelope A. Phillips–Howard, Lilian Otiso and Emanuele Giorgi in Journal of Interpersonal Violence

sj-pdf-4-jiv-10.1177_08862605221120893 – Supplemental material for Intra-Urban Variation of Intimate Partner Violence Against Women and Men in Kenya: Evidence from the 2014 Kenya Demographic and Health Survey Supplemental material, sj-pdf-4-jiv-10.1177_08862605221120893 for Intra-Urban Variation of Intimate Partner Violence Against Women and Men in Kenya: Evidence from the 2014 Kenya Demographic and Health Survey by Beate Ringwald, Rachel Tolhurst, Miriam Taegtmeyer, Lina Digolo, Grace Gichuna, Mwangi Michael Gaitho, Penelope A. Phillips–Howard, Lilian Otiso and Emanuele Giorgi in Journal of Interpersonal Violence

sj-pdf-5-jiv-10.1177_08862605221120893 – Supplemental material for Intra-Urban Variation of Intimate Partner Violence Against Women and Men in Kenya: Evidence from the 2014 Kenya Demographic and Health Survey Supplemental material, sj-pdf-5-jiv-10.1177_08862605221120893 for Intra-Urban Variation of Intimate Partner Violence Against Women and Men in Kenya: Evidence from the 2014 Kenya Demographic and Health Survey by Beate Ringwald, Rachel Tolhurst, Miriam Taegtmeyer, Lina Digolo, Grace Gichuna, Mwangi Michael Gaitho, Penelope A. Phillips–Howard, Lilian Otiso and Emanuele Giorgi in Journal of Interpersonal Violence
