# Supplementary material for: Intra-Urban Variation of Intimate Partner Violence Against Women and Men in Kenya: Evidence from the 2014 Kenya Demographic and Health Survey
Source: J Interpers Violence. 2022 Sep 5;38(5-6):5111–38. doi: 10.1177/08862605221120893 (PMC9900693; doi:10.1177/08862605221120893)
Supplement: sj-pdf-1-jiv-10.1177_08862605221120893 – Supplemental material for Intra-Urban Variation of Intimate Partner Violence Against Women and Men in Kenya: Evidence from the 2014 Kenya Demographic and Health Survey [file sj-pdf-1-jiv-10.1177_08862605221120893.pdf]

Appendix A. Characteristics of the urban female sample (age 15-49) and urban male sample (age 15-54) by residence (Kenya, 2014).

	Females					Males			
	Informal (N=608)	Intermediate (N=796)	Formal (N=209)	Total (N=1,613)		Informal (N=440)	Intermediate (N=681)	Formal (N=200)	Total (N=1,321)
**Age (years)**					**Age (years)**				
Median (IQR)	30 (25, 36)	29 (25,35)	30 (26, 35)	29 (25, 36)	Median (IQR)	35 (29, 41.25)	34 (29, 41)	35.5 (30, 42)	35 (29, 41)
**Education level**					**Education level**				
No schooling	80 (13.2%)	83 (10.4%)	4 (1.9%)	167 (10.4%)	No schooling	25 (5.7%)	24 (3.5%)	3 (1.5%)	52 (3.9%)
Primary	364 (59.9%)	348 (43.7%)	62 (29.7%)	774 (48.0%)	Primary	273 (62.0%)	267 (39.2%)	40 (20.0%)	580 (43.9%)
Secondary	150 (24.7%)	254 (31.9%)	69 (33.0%)	473 (29.3%)	Secondary	114 (25.9%)	244 (35.8%)	80 (40.0%)	438 (33.2%)
Higher	14 (2.3%)	111 (13.9%)	74 (35.4%)	199 (12.3%)	Higher	28 (6.4%)	146 (21.4%)	77 (38.5%)	251 (19.0%)
**Wealth**					**Wealth**				
Poorest	159 (26.2%)	11 (1.4%)	0 (0.0%)	170 (10.5%)	Poorest	93 (21.1%)	7 (1.0%)	0 (0.0%)	100 (7.6)
Poor	176 (28.9%)	23 (2.9%)	1 (0.5%)	200 (12.4%)	Poor	132 (30.0%)	28 (4.1%)	0 (0.0%)	160 (12.1)
Middle	138 (22.7%)	85 (10.7%)	0 (0.0%)	223 (13.8%)	Middle	126 (28.6%)	64 (9.4%)	1 (0.5%)	191 (14.5)
Rich	125 (20.6%)	304 (38.2%)	11 (5.3%)	440 (27.3%)	Rich	82 (18.6%)	265 (38.9%)	25 (12.5%)	372 (28.2)
Richest	10 (1.6%)	373 (46.9%)	197 (94.3%)	580 (36.0%)	Richest	7 (1.6%)	317 (46.5%)	174 (87.0%)	498 (37.7)
**Marital status**					**Marital status**				
Married	472 (77.6%)	615 (77.3%)	165 (78.8%)	1,252 (77.6%)	Married	373 (84.8%)	614 (90.2%)	181 (90.5%)	1,168 (88.4%)
Cohabiting	46 (7.6%)	60 (7.5%)	12 (5.7%)	118 (7.3%)	Cohabiting	15 (3.4%)	19 (2.8%)	9 (4.5%)	43 (3.3%)
Separated/ Divorced	65 (10.7%)	93 (11.7%)	29 (13.9%)	187 (11.6%)	Separated/ Divorced/ Widowed	52 (11.8%)	48 (7.0%)	10 (5.0%)	110 (8.3%)
Widowed	25 (4.1%)	28 (3.5%)	3 (1.4%)	56 (3.5%)					

**Father beat mother**					**Father beat mother**				
No	324 (53.6%)	482 (60.8%)	137 (65.6%)	947 (58.7%)	No	183 (41.8%)	360 (52.9%)	115 (57.5%)	659 (49.9%)
Don’t know	42 (6.9%)	43 (5.4%)	8 (3.8%)	93 (5.8%)	Don’t know	27 (6.1%)	37 (5.4%)	13 (6.5%)	77 (5.8%)
Yes	240 (39.5%)	269 (33.8%)	64 (30.6%)	571 (35.5%)	Yes	229 (52.0%)	284 (41.7%)	72 (36.0%)	585 (44.3%)
**Partner’s alcohol use**					**Partner’s alcohol use**				
No alcohol	396 (65.1%)	562 (70.6%)	132 (63.2%)	1,090 (67.6%)	No alcohol	413 (93.9%)	657 (96.5%)	188 (94.0%)	1,258 (95.2%)
Gets drunk often	83 (13.7%)	75 (9.3%)	31 (14.8%)	189 (11.7%)	Drinks alcohol	27 (6.1%)	24 (3.5%)	12 (6.0%)	63 (4.8%)
Gets drunk sometimes	129 (21.2%)	159 (20.0%)	45 (22.0%)	334 (20.7%)					
**Current use of physical violence against partner**					**Current use of physical violence against partner**				
No	589 (96.9%)	781 (98.1%)	202 (96.7%)	1,572 (97.5%)	No	349 (79.3%)	622 (91.3%)	178 (89.0%)	1,149 (87.0%)
Yes	19 (3.1%)	15 (1.9%)	7 (3.3%)	41 (2.5%)	Yes	91 (20.7%)	59 (8.7%)	22 (11.0%)	172 (13.0%)

Note. IQR = Inter Quartile Range.
