# Supplementary material for: Intra-Urban Variation of Intimate Partner Violence Against Women and Men in Kenya: Evidence from the 2014 Kenya Demographic and Health Survey
Source: J Interpers Violence. 2022 Sep 5;38(5-6):5111–38. doi: 10.1177/08862605221120893 (PMC9900693; doi:10.1177/08862605221120893)
Supplement: sj-pdf-2-jiv-10.1177_08862605221120893 – Supplemental material for Intra-Urban Variation of Intimate Partner Violence Against Women and Men in Kenya: Evidence from the 2014 Kenya Demographic and Health Survey [file sj-pdf-2-jiv-10.1177_08862605221120893.pdf]

Appendix B. Estimates from binomial mixed-effects models for any current intimate partner violence against women (age 15-49) and men (age 15-54) in urban areas in Kenya (2014).

	Females					Males			
	Model 1		Model 2			Model 1		Model 2	
Term	OR (95% CI)	p	aOR (95% CI)	p		OR (95% CI)	p	aOR (95% CI)	p
**Neighbourhood**					**Neighbourhood**				
Informal	1.92 (1.31, 2.83)	<0.01	1.39 (0.91, 2.13)	0.13	Informal	1.08 (0.68, 1.73)	0.73	0.89 (0.53, 1.50)	0.67
Intermediate	1.1 (0.76, 1.59)	0.62	0.94 (0.62, 1.4)	0.75	Intermediate	0.72 (0.47, 1.1)	0.13	0.79 (0.50, 1.25)	0.31
**Education level**					**Education level**				
No schooling			1.41 (0.74, 2.69)	0.29	No/ Primary/ Secondary			0.69 (0.46, 1.04)	0.08
Primary/ Secondary			2.31 (1.49, 3.57)	<0.01					
**Father beat mother**					**Father beat mother**				
Yes			1.71 (1.33, 2.2)	<0.01	Yes			1.59 (1.14, 2.22)	0.01
Don't know			1.54 (0.93, 2.56)	0.1	Don't know			1.72 (0.89, 3.31)	0.11
**Marital status**					**Marital status**				
Cohabiting			1.22 (0.78, 1.91)	0.38	Cohabiting			1.46 (0.65, 3.29)	0.36
Separated/ Divorced			1.65 (1.1, 2.49)	0.02	Separated/ Divorced/ Widowed			2.71 (1.56, 4.71)	<0.01
Widowed			0.92 (0.46, 1.83)	0.82					
**Use of physical violence against partner**					**Use of physical violence against partner**				

	Females					Males			
	Model 1		Model 2			Model 1		Model 2	
Term	OR (95% CI)	p	aOR (95% CI)	p		OR (95% CI)	p	aOR (95% CI)	p
Yes			11.32 (4.23, 30.34)	<0.01	Yes			5.76 (3.91, 8.49)	<0.01
**Partner's alcohol use**					**Partner's alcohol use**				
Sometimes drunk			1.81 (1.35, 2.42)	<0.01	Drinks alcohol			2.14 (1.11, 4.11)	0.02
Often drunk			5.86 (3.92, 8.77)	<0.01					

Note. Estimates in this table are based on binomial mixed-effects models. Any current intimate partner violence (IPV) = emotional, physical and/or sexual IPV. Residence: Reference level (Ref) = Formal; Models 1: unadjusted. Models 2: adjusted for Education (Ref=Higher), Father beat mother (Ref=No), Marital status (Ref=Married), Use of physical violence against spouse/ partner (Ref=No), Partner's alcohol use (Ref=No alcohol).
