# Supplementary material for: Intra-Urban Variation of Intimate Partner Violence Against Women and Men in Kenya: Evidence from the 2014 Kenya Demographic and Health Survey
Source: J Interpers Violence. 2022 Sep 5;38(5-6):5111–38. doi: 10.1177/08862605221120893 (PMC9900693; doi:10.1177/08862605221120893)
Supplement: sj-pdf-3-jiv-10.1177_08862605221120893 – Supplemental material for Intra-Urban Variation of Intimate Partner Violence Against Women and Men in Kenya: Evidence from the 2014 Kenya Demographic and Health Survey [file sj-pdf-3-jiv-10.1177_08862605221120893.pdf]

**Appendix C.** Estimates from binomial mixed-effects models for any current intimate partner violence against women (age 15-49) in urban areas in Kenya (2014).

| | Model 1 | | Model 2 | | Model 3 | | Model 4 | | Model 5 | | Model 6 | |
|---|---|---|---|---|---|---|---|---|---|---|---|---|
| Term | OR (95% CI) | p | aOR (95%CI) | p | aOR (95% CI) | p | aOR (95% CI) | p | aOR (95% CI) | p | aOR (95% CI) | p |
| **Neighbourhood** | | | | | | | | | | | | |
| Informal | 1.92 (1.31, 2.83) | <0.01 | 1.49 (0.99, 2.23) | 0.05 | 1.79 (1.21, 2.64) | <0.01 | 1.88 (1.28, 2.78) | <0.01 | 1.92 (1.29, 2.84) | <0.01 | 1.87 (1.25, 2.78) | <0.01 |
| Intermediate | 1.1 (0.76, 1.59) | 0.62 | 0.93 (0.64, 1.35) | 0.7 | 1.06 (0.73, 1.53) | 0.77 | 1.07 (0.74, 1.55) | 0.73 | 1.12 (0.77, 1.62) | 0.56 | 1.13 (0.77, 1.65) | 0.54 |
| **Education level** | | | | | | | | | | | | |
| No schooling | | | 1.56 (0.84, 2.91) | 0.16 | | | | | | | | |
| Primary/ Secondary | | | 2.48 (1.64, 3.74) | <0.01 | | | | | | | | |
| **Father beat mother** | | | | | | | | | | | | |
| Yes | | | | | 1.82 (1.44, 2.32) | <0.01 | | | | | | |
| Don't know | | | | | 1.45 (0.9, 2.34) | 0.12 | | | | | | |
| **Marital status** | | | | | | | | | | | | |
| Cohabiting | | | | | | | 1.19 (0.78, 1.80) | 0.42 | | | | |
| Separated/ Divorced | | | | | | | 2.29 (1.58, 3.33) | <0.01 | | | | |
| Widowed | | | | | | | 1.06 (0.55, 2.05) | 0.86 | | | | |
| **Use of physical violence against partner** | | | | | | | | | | | | |
| Yes | | | | | | | | | 12.17 (4.66, 31.77) | <0.01 | | |
| **Partner's alcohol use** | | | | | | | | | | | | |
| Sometimes drunk | | | | | | | | | | | 1.88 (1.42, 2.49) | <0.01 |
| Often drunk | | | | | | | | | | | 6.55 (4.44, 9.65) | <0.01 |

Note. Estimates in this table are based on binomial mixed-effects models. Any current intimate partner violence (IPV) = emotional, physical and/or sexual IPV. Residence: Reference level (Ref) = Formal; Model 1: unadjusted. Model 2: adjusted for Education attainment (Ref=Higher); Model 3: adjusted for Father beat mother (Ref=No); Model 4: adjusted for Marital status (Ref=Married); Model 5: adjusted for Use of physical violence against spouse/ partner (Ref=No); Model 6: adjusted for Partner's alcohol use (Ref=No alcohol).
