# Supplementary material for: Intra-Urban Variation of Intimate Partner Violence Against Women and Men in Kenya: Evidence from the 2014 Kenya Demographic and Health Survey
Source: J Interpers Violence. 2022 Sep 5;38(5-6):5111–38. doi: 10.1177/08862605221120893 (PMC9900693; doi:10.1177/08862605221120893)
Supplement: sj-pdf-4-jiv-10.1177_08862605221120893 – Supplemental material for Intra-Urban Variation of Intimate Partner Violence Against Women and Men in Kenya: Evidence from the 2014 Kenya Demographic and Health Survey [file sj-pdf-4-jiv-10.1177_08862605221120893.pdf]

Appendix D. Estimates from binomial mixed-effects models for any current intimate partner violence against men (age 15-54) in urban areas in Kenya (2014).

	Model 1		Model 2		Model 3		Model 4		Model 5		Model 6	
Term	OR (95% CI)	p	aOR (95%CI)	p	aOR (95% CI)	p	aOR (95% CI)	p	aOR (95% CI)	p	aOR (95% CI)	p
**Neighbourhood**												
Informal	1.08 (0.68, 1.73)	0.73	1.19 (0.73, 1.95)	0.48	1.01 (0.63, 1.61)	0.97	1.04 (0.65, 1.67)	0.87	0.87 (0.54, 1.41)	0.58	1.08 (0.68, 1.73)	0.74
Intermediate	0.72 (0.47, 1.1)	0.13	0.75 (0.49, 1.17)	0.21	0.7 (0.46, 1.09)	0.11	0.71 (0.46, 1.1)	0.13	0.73 (0.47, 1.13)	0.16	0.74 (0.48, 1.14)	0.17
**Education level**												
No/Primary/ Secondary			0.77 (0.53, 1.13)	0.18								
**Father beat mother**												
Yes					1.69 (1.23, 2.32)	<0.01						
Don't know					1.99 (1.09, 3.66)	0.03						
**Marital status**												
Cohabiting							1.35 (0.62, 2.93)	0.45				
Separated/ Divorced/ Widowed							2.3 (1.38, 3.84)	<0.01				
**Use of physical violence against partner**												
Yes									5.71 (3.92, 8.31)	<0.01		
**Partner's alcohol use**												
Yes											2.9 (1.6, 5.23)	<0.01

Note. Estimates in this table are based on binomial mixed-effects models. Any current intimate partner violence (IPV) = emotional, physical and/or sexual IPV. Residence: Reference level (Ref) = Formal; Model 1: unadjusted. Model 2: adjusted for Education attainment (Ref=Higher); Model 3: adjusted for Father beat mother (Ref=No); Model 4: adjusted for Marital status (Ref=Married); Model 5: adjusted for Use of physical violence against spouse/ partner (Ref=No); Model 6: adjusted for Partner's alcohol use (Ref=No alcohol).
