# Supplementary material for: Intra-Urban Variation of Intimate Partner Violence Against Women and Men in Kenya: Evidence from the 2014 Kenya Demographic and Health Survey
Source: J Interpers Violence. 2022 Sep 5;38(5-6):5111–38. doi: 10.1177/08862605221120893 (PMC9900693; doi:10.1177/08862605221120893)
Supplement: sj-pdf-5-jiv-10.1177_08862605221120893 – Supplemental material for Intra-Urban Variation of Intimate Partner Violence Against Women and Men in Kenya: Evidence from the 2014 Kenya Demographic and Health Survey [file sj-pdf-5-jiv-10.1177_08862605221120893.pdf]

Appendix E. Variance of the random effects

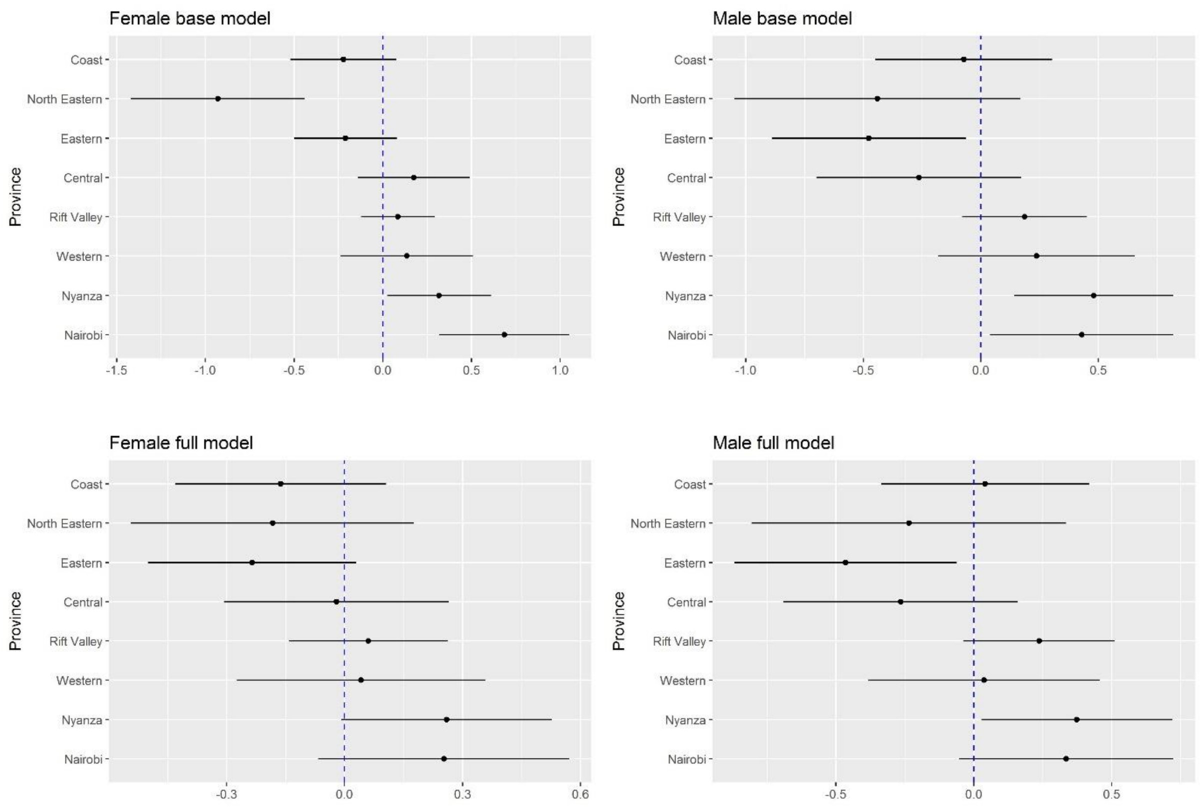

Note. Binomial mixed-effects models for any current intimate partner violence against women (age 15-49) and men (age 15-54) in urban areas in Kenya (2014). Random variable = Province. Any current intimate partner violence (IPV) = emotional, physical and/or sexual IPV.
